# Biomarker Identification from RNA-Seq Data using a Robust Statistical Approach

**DOI:** 10.6026/97320630014153

**Published:** 2018-04-30

**Authors:** Zobaer Akond, Munirul Alam, Md. Nurul Haque Mollah

**Affiliations:** 1 Agricultural Statistics and Information & Communication Technology (ASICT) Division, Bangladesh Agricultural Research Institute (BARI), Joydebpur, Gazipur-1701, Bangladesh; 2 Institute of Environmental Science, University of Rajshahi-6205, Bangladesh; 3 Emerging Infections, Infectious Diseases Division, International Centre for Diarrheal Disease Research, Bangladesh (icddr,b); 4 Bioinformatics Lab, Department of Statistics, University of Rajshahi, Rajshahi-6205, Bangladesh

**Keywords:** RNA-seq data, differentially expressed genes, robust t-statistic, gene-disease network, protein-protein interaction

## Abstract

Biomarker identification by differentially expressed genes (DEGs) using RNA-sequencing technology is an important task to characterize the transcriptomics data. This is possible with the advancement of next-generation sequencing technology (NGS). There are a number of statistical techniques to identify DEGs from high-dimensional RNA-seq count data with different groups or conditions such as edgeR, SAMSeq, voom-limma, etc. However, these methods produce high false positives and low accuracy in presence of outliers. We describe a robust t-statistic method to overcome these drawbacks using both simulated and real RNA-seq datasets. The model performance with 61.2%, 35.2%, 21.6%, 6.9%, 74.5%, 78.4%, 93.1%, 35.2% sensitivity, specificity, MER, FDR, AUC, ACC, PPV, and NPV, respectively at 20% outliers is reported. We identified 409 DE genes with p-values<0.05 using robust t-test in HIV viremic vs avirmeic state real dataset. There are 28 up-regulated genes and 381 down-regulated genes estimated by log2 fold change (FC) approach at threshold value 1.5. The up-regulated genes form three clusters and it is found that 11 genes are highly associated in HIV- 1/AIDS. Protein-protein interaction (PPI) of up-regulated genes using STRING database found 21 genes with strong association among themselves. Thus, the identification of potential biomarkers from RNA-seq dataset using a robust t-statistical model is demonstrated.

## Background

Transcriptomics is an evolving and continually growing field of Bioinformatics for biomarker discovery [1]. RNA-sequencing (RNA-seq) technology is an important branch of transcriptomics for identification of novel genes. This technique helps to identify the differentially expressed genes or transcripts (DEGs) associated to trait of interest from the voluminous transcriptomic data. Previously microarray technology had been used by the biological and biomedical researchers for discovering the candidate genes and differentially expressed markers between two or more groups of interest. In recent years, huge amount of transcriptomic data can be generated using high-throughput next-generation sequencing (NGS) technology of cDNA (RNAseq), which ultimately yield RNA-seq count data for subsequent analysis [6]. Additionally, this approach includes the identification of disease biomarkers that may be important in the diagnosis of the different types and subtypes of diseases, with several implications in terms of prognosis and therapy [2]. This sequence-based technology has created significant scope of studying the transcriptome by enabling a wide range of novel applications, including detection of alternative splicing isoforms [3, 4], detecting novel genes, gene promoters, isoforms, and allelespecific expression [5]. RNA-seq uses NGS technology to sequence cDNA that has been derived from an RNA sample, and hence generates millions of short reads [6]. These reads are then usually mapped to a reference genome and the number of reads mapping within a genomic feature of interest (such as a gene or an exon) is used as a measure of the abundance of the feature in the analyzed sample [6]. One important objective for RNA-seq is to identify DEGs under different conditions. Researchers typically target for differential expression analysis called "count matrix", where each row represents the gene (or exons or genomic loci), each column represents the sample, and each cell indicates the number of reads mapped to the gene in the sample [7]. A basic research problem in many RNA-seq analyses is the discovery of DEGs between different sample groups (e.g. healthy and disease). RNA-seq analysis has some benefits over microarrays for DE analysis including wide dynamic range and a lower background level, and the chance to detect and quantify the expression of previously unknown transcripts [8, 9, 10]. To deal with the increasing popularity of RNA-seq technology, several statistical tools have been developed so far by the researchers and these are being continuously updated for robust data analysis. Most of the computational methods such as edgeR [11] are based on negative binomial models. SAMseq [12] is a non-parametric method. Gene-level read counts transformation-based method is voom-limma [14]. Still it is important to keep in mind that even these methods are based on an assumption that most genes are equally expressed in the samples, and that the differentially expressed genes are divided more or less equally between upand down-regulation [2]. For detection of DE genes edgeR, SAMSeq and voom-limma are the popular methods. However these methods are sensitive to outliers. In this study we propose a new method robust t-statistic using minimum β-divergence method [15].

## Methodology

RNA-seq count data often produce noisy data called outliers during data generating steps. Presence of such outliers in the particular dataset will surely provide misleading results in the downstream analysis. There is however a number of statistical tools for identification of over-expressed and under-expressed genes, but they are sensitive to outliers in some cases. In this investigation we considered edgeR, SAMSeq and voom-limma the proposed procedures for performance analysis with the help of synthetic and real dataset (Figure 8).

### Robust t-statistic for Biomarker Detection (Proposed)

The two sample-sample t-test statistic depends on the mean (μ) and variance (σ2) of the samples. The classical mean and variance are very much sensitive to outliers may mislead detection of DEGs. So, we used robust mean and variance with the minimum β-divergence method [15] instead of classical estimators. For robust t-statistic the classical estimators are replaced by the β- estimators. The proposed method tolerate outliers using the β- weight function, it is also used as a weight for mean and variance estimators [15]. It is an iterative method for estimating mean and variance estimators for calculating t-statistic values. The performance of the proposed method depends on the tuning parameter β, the optimum value for β=0.2 was selected using cross validation method [15].

### Data Transformation

**Figure 1:**
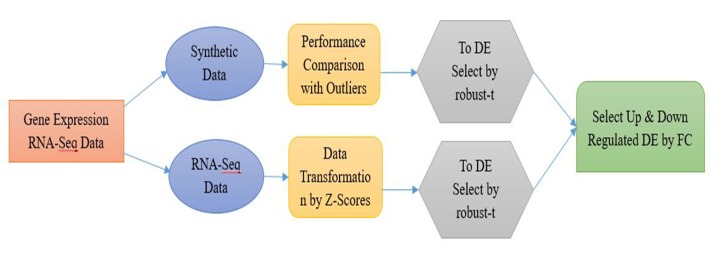
Block diagram for proposed method

To transform the RNA-Seq expression data we used the z- score transformation for making the data belong normalized data family. In this study Z normalized score values were used for the normalization with the mean (g) and the standard deviation SD (gi) of the ith genes. The normalized data is used to detect the DE genes using robust t-statistic. The fold change (FC) log2 approach used for selection of up-regulated and down-regulated genes with 1.5 threshold value [16]. The steps for identifying up and down regulated genes from gene expression data (GED) are shown (Figure 1).

### Performance Evaluation

We need statistical indices/measures to estimate the performance of different DE gene identification methods for binary classification tests such as healthy and disease. Outcomes are always divided into four categories for binary classification such as (i) healthy samples are correctly predicted as healthy termed as true positive (TP), (ii) healthy sample are incorrectly predicted as disease termed as false negative (FN), (iii) disease samples are correctly predicted as disease termed as true negative (TN) and (iv) disease samples are incorrectly predicted as healthy which is called false positive (FP) in Table 1. We then calculate the following performance indices/measures using the following confusion matrix as follows: True Positive Rate (TPR) or Sensitivity = nTP/(nTP+nFN), True Negative Rate (TNR) or Specificity = nTN/(nTN+nFP), False Positive Rate (FPR) = nFP/(nFP+nTN), False Negative Rate (FNR) = nFN/(nFN+nTP) and False Discovery Rate (FDR)= nFP/(nTP+nFP).

## Results and Discussion

All methods were run on the same dataset and evaluated the statistical performance indices/measures such as ROC, AUC, pAUC, MER and FDR. The performance measure AUC was computed for each of the statistical methods using open source R package ROC. All R packages are freely available in the comprehensive R archive network (https://www.cran.rproject. org) and bioconductor (https://www.bioconductor.org).

### Synthetic Study

**Figure 2:**
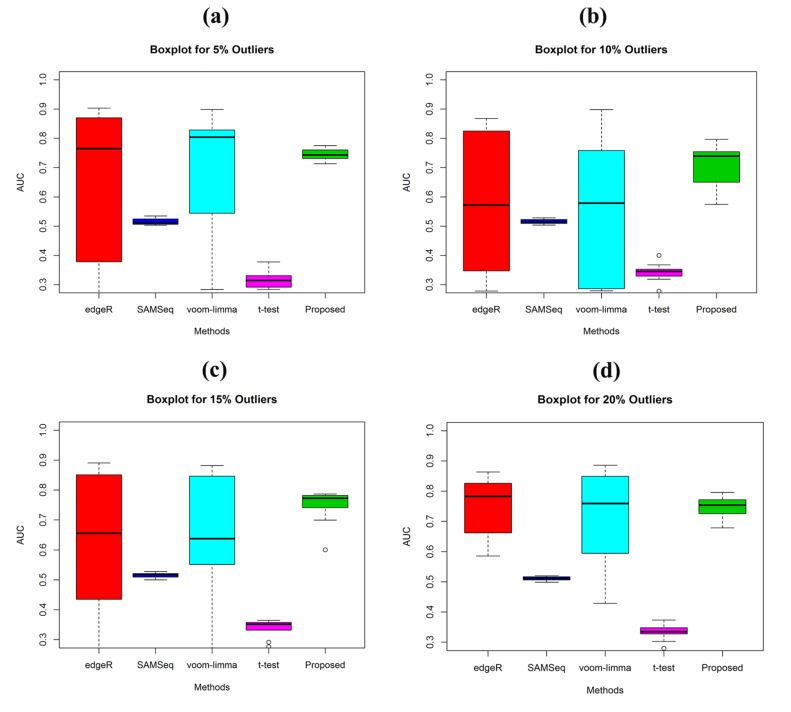
Performance investigation using the boxplot of AUC values of the four methods (edgeR, SAMSeq, voom-limma and proposed) for sample sizes (n1=n2=3). (a) In presence of 5% outliers (b) In presence of 10% outliers (c) In presence of 15% outliers (d) In presence of 20% outliers.

**Figure 3:**
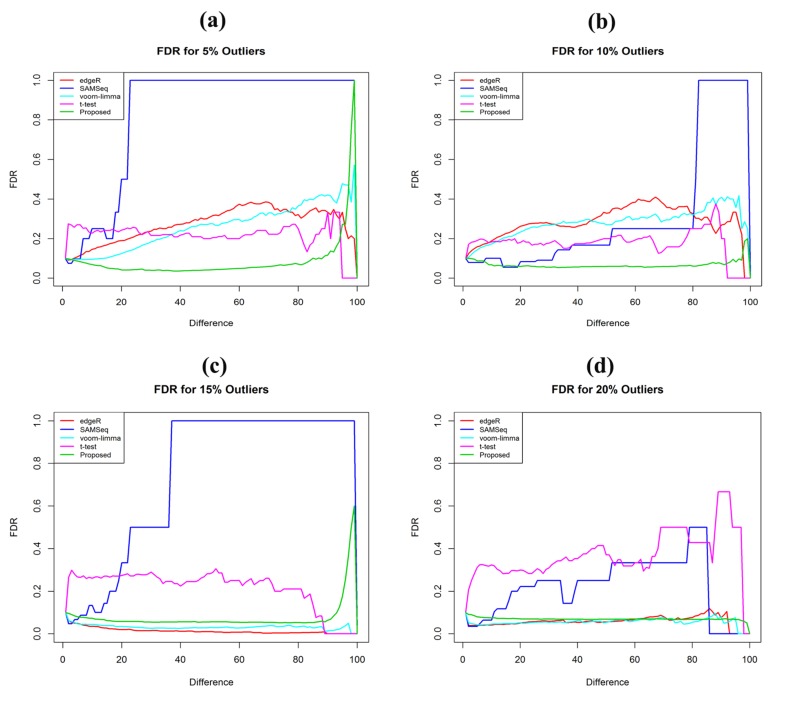
Performance test using FDR plot of the four methods (edgeR, SAMSeq, voom-limma and proposed) for sample sizes (n1=n2=3). (a) In presence of 5% outliers (b) In presence of 10% outliers (c) In presence of 15% outliers (d) In presence of 20% outliers

**Figure 4:**
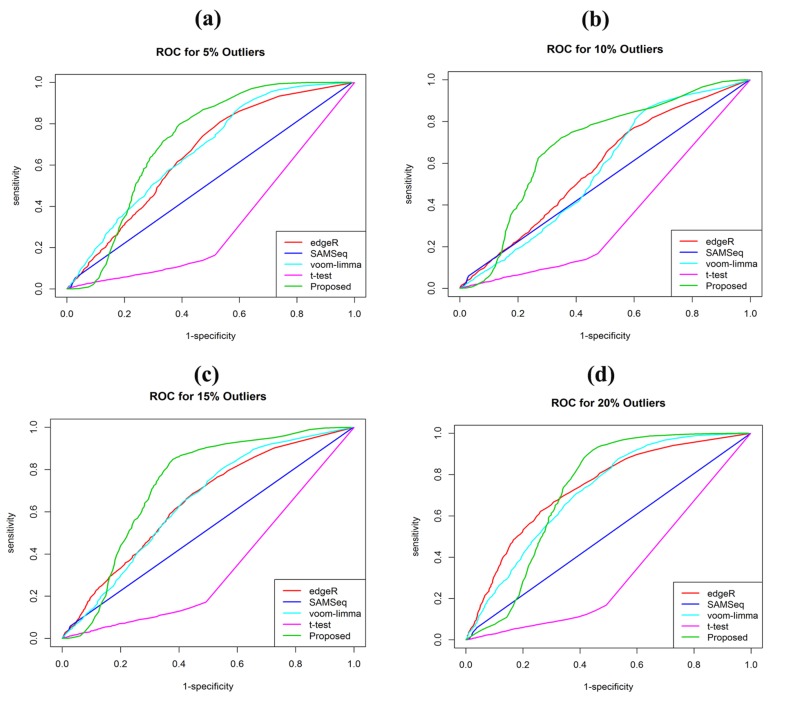
Performance analysis using the ROC of the four methods (edgeR, SAMSeq, voom-limma and proposed). (a) In presence of 5% outliers (b) In presence of 10% outliers (c) In presence of 15% outliers (d) In presence of 20% outliers.

The synthetic counts data were generated for each gene from a Negative Binomial distribution. It controls the settings and the true differential expression status of each gene. We generated gene expression profiles of G=1000 genes of k=2 groups (healthy and disease) each with n1=n2=3 samples. Among the expressions of 1000 genes, we divided these expressions into two groups (expressions of important genes or DE genes, 100 and expressions of the unimportant genes or EE genes, 900). In order to determine the performance of the robust t-test in comparison of the three well-known DE genes identification methods (edgeR, SAMSeq, voom+limma) of RNA-Seq data, we investigated the performance of all fours methods in presence of outliers using the simulated dataset (G=1000) where 10% of the genes are DE genes. A method is said to be comparatively good performer if it yields larger values of TPR, TNR, AUC and small value of FDR. To demonstrate the effect in presence of outliers for performance evaluation of these DE genes identification methods, we randomly introduce 5%, 10%, 15% and 20% genes are corrupted by outliers. (Figure 2a-d) shows the boxplot of AUC values of the four methods estimated from the simulated data at different outlier levels. It showed that the AUC of our proposed method is high. It is observed from the Table 2 that the AUC values of proposed method are 0.75, 0.71, 0.74 and 0.75 for 5%, 10%, 15% and 20% respectively. The proposed method provided the low FDR and high ROC with outliers (Figure 3 and Figure 4). Identification of DEGs is vital issues for personalised medicine and drug discovery. From Table 2 the proposed method shows low MER (misclassification error rate) than the edgeR, SAMSeq and voomlimma methods.

### Real RNA-Seq Data Analysis

**Figure 5:**
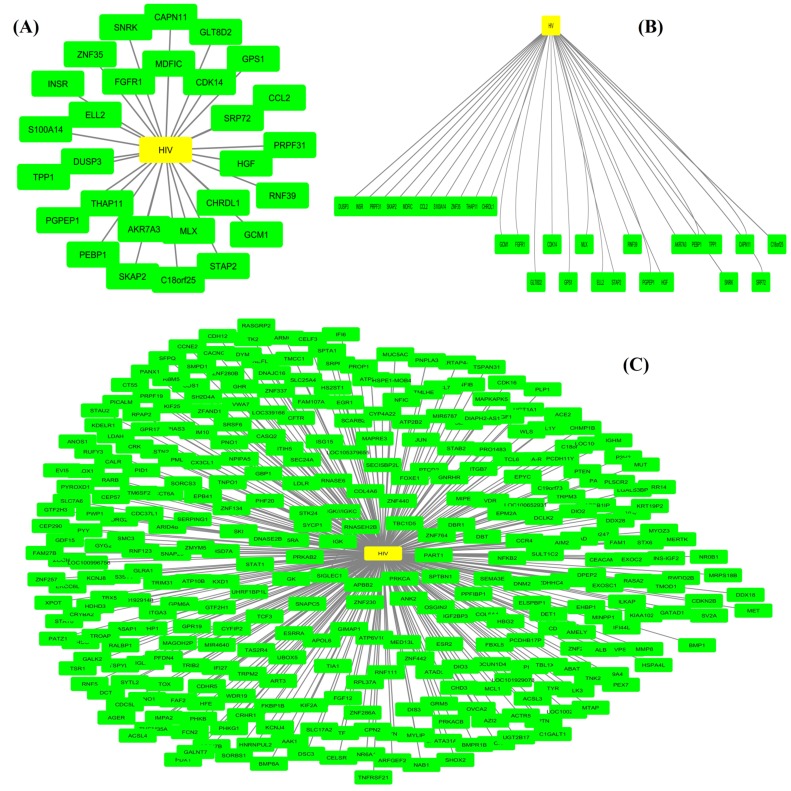
(A) Up-regulated gene-disease network (B) Hierarchical clustering of up-regulated genes (C) Gene-disease network of downregulated genes.

**Figure 6:**
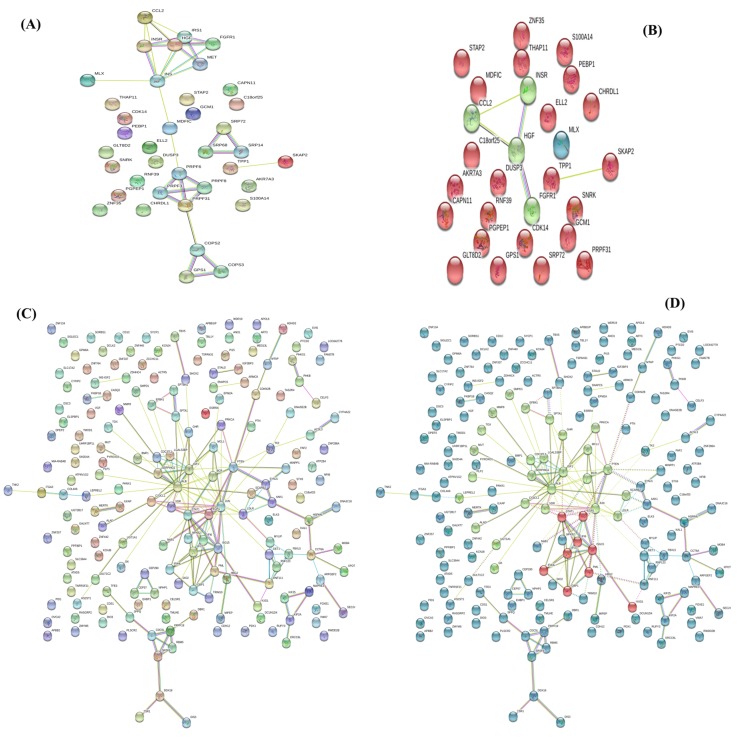
(A) Up-regulated gene protein-protein interaction (PPI) network (B) K-mean clustering of up-regulated genes (C) Downregulated gene PPI networks and (D) K-means clustering of down-regulated genes of HIV disease.

**Figure 7:**
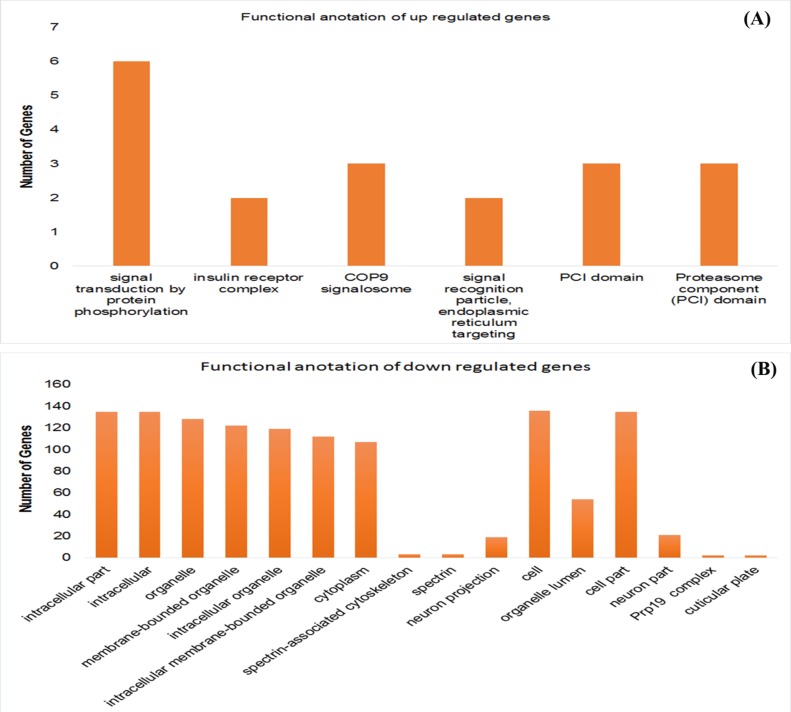
Functional annotation of (A) Up-regulated DE genes (B) Down-regulated genes of real RNA-Seq HIV disease dataset.

**Figure 8:**
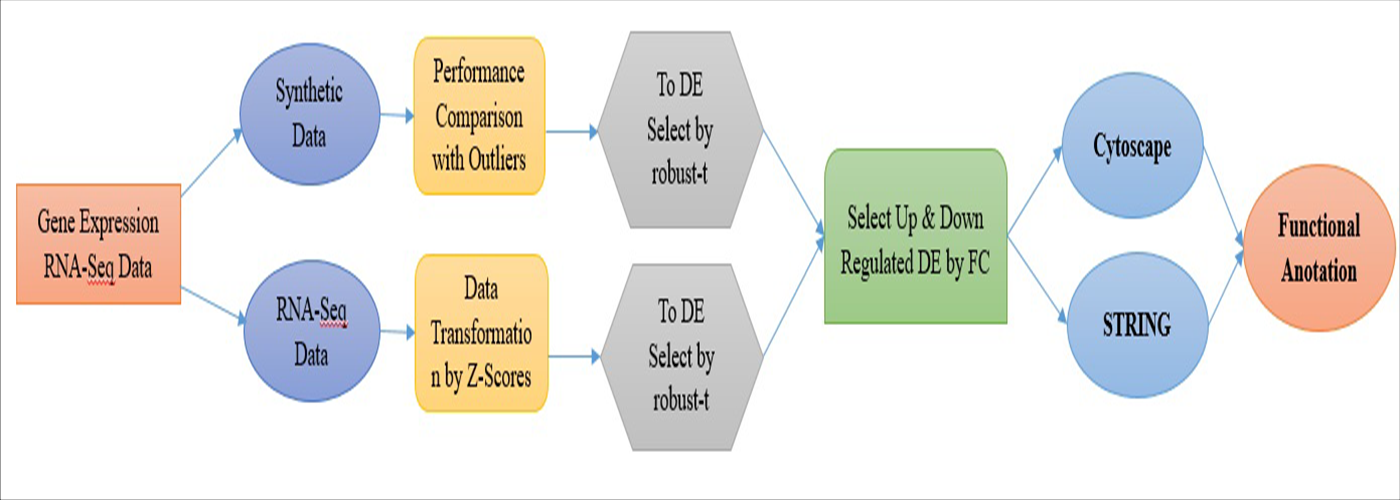
Full work flow of the study paper

The RNA-Seq data GSE5220 was collected from the GEO database (https://www.ncbi.nlm.nih.gov/geo/query/acc.cgi?acc=GSE522 0) [17]. It contains 16 HIV infected patients (8 patients are pre and 8 are post HAART cessation) and 22283 genes. It is observed that the gene expression changes in monocytes as a result of rebound of HIV viral load. From the HIV viremic vs avirmeic state real dataset we identified 409 DE genes at 5% level of significance with p-values<0.05 using robust t-test. There are 28 up- regulated genes and 381 down regulated genes by log2 fold change (FC) approach with threshold value 1.5 from the RNA-Seq count data [16]. The molecular networks of the selected up-regulated genes are visualized using open source bioinformatics software platform Cytoscape (version 3.6.0) [18], the gene-disease network shows the interaction among genes and HIV disease. Figure 5a showed that there are 11 up-regulated genes (MDFIC, CDK14, SRP72, HGF, CHRDL1, MLX, AKR7A3, THAP11, DUSP3, ELL2, and FGFR1), which are highly correlated that are more interaction is existed with the HIV than the other genes. Figure 5b showed the hierarchical clustering of 28 up-regulated genes, which formed 3 different gene major functional groups. The group-I (DUSP3, INSR, PRPF31, SKAP2, MDFIC, CCL2, SIOOA14, ZNF35, THAP11, CHRDL1) genes are significant for the biological process in the cell, group-II genes (GCM1, FGFR1, GLT8D2, CDK14, G PS1, MLX, ELL2, STAP2, RNF39, PGPEP1, HGF) are insulin receptor complex, COP9 signalosome and insulin receptor complex and group-III genes (AKR74A3, PEBPI, TPP1, SNRK, CAPN11, SRP72, C18orf25) are proteosome component (PCI) domain composition. From the Figure 5c the 381 down-regulated genes are detected by the proposed method. There are 10 genes (IGK, PRKAB2, SIGLEC1, APBB2, PRKCA, SPTBN1, PART1, ZNF764, TBC1D5, and RNASEH2B), which are highly correlated with the HIV disease and possess strong interaction with HIV disease. The protein-protein interaction of up-regulated genes analysis using STRING (version 10.5) database [19] produced three functional clustering groups (Figure 6a-b) using k-means clustering algorithm. There are 21 genes showed the strong association among them (Figure 6a) and 20 genes are structured proteins. The MDFIC gene is the unstructured protein and it helps to make strong bond among other genes. The PPI network among 381 down-regulated genes is strong which forms three functional groups using k-means clustering algorithm (Figure 6c-d). There are 45 unstructured genes whose protein structures are not available. The functional annotation of up-regulated and down-regulated genes is shown in the Figure 7a-b respectively. The details of their cellular component functional annotations are shown in Table 3.

## Conclusions

RNA-seq data analysis helps to identify DEGs to solve important biological problems. Statistical approaches such as edgeR, SAMSeq and voom-limma are widely used in DEGs identification. However, these methods are sensitive to outliers. Hence, we report a robust t-statistic using minimum β-divergence method to overcome this issue. Analysis of synthetic data showed that the robust t-statistic method produced better performance with high AUC, low MER and FDR. The real RNAseq count data analysis detected 409 DEG using this method. There are 28 up-regulated genes and 381 down-regulated genes estimated by log2 fold change (FC) approach at threshold value 1.5. Thus, the identification of potential biomarkers from RNAseq dataset using a robust t-statistical model is demonstrated.

## Figures and Tables

**Table 1:** Confusion matrix or error matrix

Predicted class	Actual class	
DE	EE
DE	True Positive (TP)	False Positive (FP) type I error
EE	False Negative (FN) (type II error)	True Negative (TN)
DE: Differentially Expressed; EE: Equally Expressed; Area under the receiving operating characteristics (ROC) curve, AUC = (nTPR+nTNR)/2, Misclassification error rate (MER) = (nFP+nFN)/ (nTP+nTN+nFP+nFN).		

**Table 2:** Performance Investigation for synthetic dataset

Performance Evaluation	edgeR	SAMSeq	Voom.limma	t-test	Proposed
5% outliers					
Sensitivity	0.36	0.015	0.493	0.046	0.546
Specificity	0.761	0.984	0.325	0.046	0.314
MER	0.774	0.89	0.691	0.867	0.537
FDR	0.269	0.821	0.256	0.216	0.085
AUC	0.664	0.515	0.694	0.32	0.744
pAUC	0.057	0.009	0.059	0.006	0.024
ACC	0.226	0.11	0.309	0.133	0.463
PPV	0.731	0.179	0.744	0.784	0.915
NPV	0.761	0.984	0.325	0.046	0.314
10% outliers					
Sensitivity	0.372	0.019	0.391	0.046	0.476
Specificity	0.693	0.982	0.346	0.046	0.27
MER	0.812	0.884	0.796	0.859	0.685
FDR	0.28	0.314	0.276	0.176	0.066
AUC	0.581	0.515	0.57	0.342	0.71
pAUC	0.048	0.01	0.036	0.006	0.024
ACC	0.188	0.116	0.204	0.141	0.315
PPV	0.72	0.686	0.724	0.824	0.934
NPV	0.693	0.982	0.346	0.046	0.27
15% outliers					
Sensitivity	0.364	0.018	0.421	0.048	0.64
Specificity	0.764	0.985	0.291	0.048	0.342
MER	0.445	0.889	0.47	0.869	0.227
FDR	0.014	0.732	0.0323	0.213	0.075
AUC	0.634	0.516	0.642	0.337	0.748
pAUC	0.055	0.01	0.047	0.007	0.029
ACC	0.555	0.111	0.53	0.131	0.773
PPV	0.986	0.268	0.9677	0.787	0.925
NPV	0.764	0.985	0.291	0.048	0.342
20% outliers					
Sensitivity	0.405	0.017	0.439	0.046	0.612
Specificity	0.812	0.979	0.247	0.046	0.352
MER	0.702	0.885	0.652	0.878	0.216
FDR	0.058	0.217	0.055	0.366	0.069
AUC	0.742	0.51	0.724	0.333	0.745
pAUC	0.064	0.01	0.053	0.006	0.022
ACC	0.298	0.115	0.348	0.122	0.784
PPV	0.942	0.783	0.945	0.634	0.931
NPV	0.812	0.979	0.247	0.046	0.352

**Table 3:** Functional annotation of down-regulated genes

Cellular Component (GO)			
Pathway ID	Pathway Description	Count in gene set	False discovery rate (FDR)
GO:0044424	Intracellular part	135	8.95E-05
GO:0005622	Intracellular	135	0.000353
GO:0043226	Organelle	128	0.000572
GO:0043227	Membrane-bounded organelle	122	0.00147
GO:0043229	Intracellular organelle	119	0.0018
GO:0043231	Intracellular membrane-bounded organelle	112	0.00282
GO:0005737	Cytoplasm	107	0.00832
GO:0014731	spectrin-associated cytoskeleton	3	0.00832
GO:0008091	spectrin	3	0.00984
GO:0043005	Neuron projection	19	0.0146
GO:0005623	Cell	136	0.023
GO:0043233	Organelle lumen	54	0.023
GO:0044464	Cell part	135	0.0298
GO:0097458	Neuron part	21	0.0307
GO:0000974	Prp19 complex	2	0.0439
GO:0032437	cuticular plate	2	0.0439

## References

[1] David S Biomarkers in Toxicology..

[2] Dillies MA (2012). Briefings in Bioinformatics..

[3] Wang (2008). Nature..

[4] Pan Q (2008). Nature Genetics..

[5] Landau (2013). PLoS ONE..

[6] Soneson C, Delorenzi MA. (2013). BMC Bioinformatics..

[7] Tang (2015). BMC Bioinformatics..

[8] Oshlack A (2010). Genome Biology..

[9] Agarwal (2010). BMC Genomics..

[10] Bradford JR (2010). BMC Genomics..

[11] Robinson MD (2010). Bioinformatics..

[12] Li J, Tibshirani R. (2013). Statistical Methods in Medical Research..

[13] Ander S, Huber W. (2010). Genome Biology..

[14] Smyth GK. (2004). Statistical Applications in Genetics and Molecular Biology..

[15] Mollah (2010). Neural Networks..

[16] Dembélé D, Kastner P. (2014). BMC Bioinformatics..

[17] Tilton JC (2006). Journal of Virology..

[18] Shannon P (2003). Genome Research..

[19] Szklarczyk D (2017). Nucleic Acids Research..

